# Community health center provider ability to identify, treat and account for the social determinants of health: a card study

**DOI:** 10.1186/s12875-016-0526-8

**Published:** 2016-08-27

**Authors:** Joy H. Lewis, Kate Whelihan, Isaac Navarro, Kimberly R. Boyle, SDH Card Study Implementation Team

**Affiliations:** 1 A.T. Still University of Health Sciences School of Osteopathic Medicine in Arizona (ATSU SOMA), 5850 E. Still Circle, Mesa, AZ 85206 USA; 2 KRB Clinical Solutions LLC, 24 Underwood Road, Montville, NJ 07045 USA

**Keywords:** Social determinants of health, Primary care, Health care providers, Community Health Centers, Electronic Health Record, Diagnosis codes, Procedure codes, ICD-10

## Abstract

**Background:**

The social determinants of health (SDH) are conditions that shape the overall health of an individual on a continuous basis. As momentum for addressing social factors in primary care settings grows, provider ability to identify, treat and assess these factors remains unknown. Community health centers care for over 20-million of America’s highest risk populations. This study at three centers evaluates provider ability to identify, treat and code for the SDH.

**Methods:**

Investigators utilized a pre-study survey and a card study design to obtain evidence from the point of care. The survey assessed providers’ perceptions of the SDH and their ability to address them. Then providers filled out one anonymous card per patient on four assigned days over a 4-week period, documenting social factors observed during encounters. The cards allowed providers to indicate if they were able to: provide counseling or other interventions, enter a diagnosis code and enter a billing code for identified factors.

**Results:**

The results of the survey indicate providers were familiar with the SDH and were comfortable identifying social factors at the point of care. A total of 747 cards were completed. 1584 factors were identified and 31 % were reported as having a service provided. However, only 1.2 % of factors were associated with a billing code and 6.8 % received a diagnosis code.

**Conclusions:**

An obvious discrepancy exists between the number of identifiable social factors, provider ability to address them and documentation with billing and diagnosis codes. This disparity could be related to provider inability to code for social factors and bill for related time and services. Health care organizations should seek to implement procedures to document and monitor social factors and actions taken to address them. Results of this study suggest simple methods of identification may be sufficient. The addition of searchable codes and reimbursements may improve the way social factors are addressed for individuals and populations.

**Electronic supplementary material:**

The online version of this article (doi:10.1186/s12875-016-0526-8) contains supplementary material, which is available to authorized users.

## Background

Public health officials have long recognized social and environmental factors play a role in impacting an individual’s health. In fact, the social determinants of health (SDH) have been an ever present theme in the works and initiatives set forth by the World Health Organization (WHO) since its inception in 1948 [1]. Defined by the WHO as “the conditions in which people are born, grow, live, work, and age” [2] the social determinants of health are conditions that shape the overall health of an individual on a continuous basis.

A significant upswing in momentum for SDH focus was the development of the WHO’s Commission on Social Determinants of Health on March 18, 2005 [3]. The commission expands on the WHO definition of SDH and states that “avoidable health inequalities arise because of the circumstances in which people grow, live, work, and age, *AND* the systems put in place to deal with illness” (emphasis added) [4]. Identifying social factors and relating them to health conditions for individuals and for populations is of the utmost importance. This importance has been further emphasized by the Institute of Medicine (IOM) with the development of a framework for educating health professionals to address the social determinants of health [5].

Despite the increased focus on SDH, the US suffers from relatively poor health status and worse health outcomes when compared to other high-income nations. SDH can both directly affect health and limit the ability of individuals to make healthy choices [6]. SDH are commonly considered factors associated with economic stability, neighborhood and physical environment, education, food and nutrition, social contexts and health care access [7]. Specific examples of SDH include substandard education, food insecurity, unstable housing and unsafe neighborhoods, environmental conditions and low income [8, 9]. The evidence base for the impact of these factors on health outcomes is growing. Chronic diseases such as diabetes, cardiovascular disease and asthma can be linked to social determinants such as low income and education level [10]. Food insecurity puts people at an increased risk for hypertension, hyperlipidemia and diabetes [11, 12].

The “American Health Care Paradox” - an unfavorable balance between health care costs and health of the population [13] - may be evidence of the lack of attention health care providers give to SDH, despite increasing evidence which suggests that SDH impact health conditions more than the care provided in medical offices [13]. Attention is spent at the point of care to evaluate and motivate patients towards positive health behaviors. However, without addressing social factors, it is difficult to affect positive change. In 2008, Forde and Raine mention, “social factors such as poverty and its sequelae substantially affect people’s abilities to adopt healthy behaviors” [14].

The existence of SDH and the health inequities they create are estimated to be responsible for 30 % of direct medical excess costs for Blacks, Hispanics and Asian Americans [15]. In an increasingly diverse population, efforts to address the SDH are essential to improving quality of care and health outcomes [15]. A study by Milstein, et al. points out that only when a multi-layered approach to health care improvement is completed will the result of better outcomes along with lower cost be achieved [16].

The implementation of the Patient Protection and Affordable Care Act (ACA) will allow for many factors impacting the overall health of the US population to be addressed. The ACA will allow for widespread dissemination of preventive practices that should take SDH into account. The Prevention and Public Health Fund will assure preventive practices related to housing, transportation, food security, and environment while the expansion of Medicaid and the advent of the Health Exchange Marketplace will help assure widespread access to necessary health care delivery [17]. This multifactorial approach has the potential to make a profound impact on the overall health status of the average US citizen.

To address the increased need for SDH services in health care, the Institute of Medicine’s Committee on the Recommended Social and Behavioral Measures for Electronic Health Records (EHR) was convened to develop measures for SDH that could be incorporated into EHRs [18]. They were also tasked with minimizing the barriers to the use of these measures [18]. The committee recognized the importance of addressing SDH in clinical practice and the need for an efficient, systematic way to do so. They prioritized SDH with the “greatest clinical usefulness and feasibility for capture in the clinical workflow” [10]. The panel of SDH measures they developed is intended to be brief and readily adoptable into any EHR system to allow clinicians everywhere to incorporate these measures into the treatment of all patients [10].

The ability to treat the SDH is particularly important for providers at Community Health Centers (CHCs). In the United States, CHCs provide primary health care services for nearly 22 million of the nation’s poorest and highest risk individuals [18–20]. CHCs are primary health care organizations typically located in designated Medically Underserved Areas. Governed by a board of directors consisting of local community members with a makeup of at least 51 % health center patients, these organizations provide comprehensive services including enabling services such as education, translation, and transportation. CHCs are ‘safety net’ organizations; as such they provide services to all community members regardless of ability to pay [21]. Over 70 % of health center patients have a family income at or below poverty. A reported 38 % of health center patients are uninsured and 39 % have Medicaid as their insurance [22]. CHCs also serve many who would likely not have other access to care such as rural populations, migrant workers and the homeless [23].

Most CHCs have programs focused on helping patients with some aspects of the SDH, but it is not clear if all CHC primary care providers understand the relationship among the programs, SDH and patient health [24]. Most providers likely recognize social factors but they do not necessarily understand the relationship between these factors and health outcomes. Providers may not be confident regarding how to address the SDH [25]. Additionally, it is not clear if CHC providers are able to code for their provision of services to address specific SDH or if they are able to utilize diagnosis codes for identified SDH.

With the current atmosphere related to the expansion of health care coverage under the ACA and with new opportunities for CHCs to refine the methods in which they service their communities, now is the time for CHCs to review their existing methods and make necessary changes. A first step to achieving this change is a better understanding of the specific issues faced by patients who visit CHCs and the way in which these issues are addressed.

This study was a collaboration involving multiple CHCs in varying locations (CA, NY and IL) in order to record SDH factors determined by providers to be affecting patients and to evaluate provider perceptions of the impact of these factors on their patients’ health. Further, this study aimed to assess providers’ abilities to address, bill for services focused on the SDH and code for identified SDH. Ultimately, this study sought to document specific information about what is happening, related to the SDH, at the point of care at varying CHC locations. The ultimate intent was to inform policy.

## Methods

The A.T. Still University School of Osteopathic Medicine in Arizona Practice Based Research Network (ATSU SOMA PBRN) implemented a card study from May, 2014 – December, 2015 related to the SDH. The ATSU SOMA PBRN is made up of primary care providers at 12 CHCs across the United States [26]. The PBRN mission is directly related to this project: “focusing on the social determinants of health and the needs of America’s underserved and vulnerable populations we will conduct clinically relevant research to develop and share sustainable programs to improve health care access and quality” [26]. This project was reviewed and deemed exempt by the ATSU Arizona Institutional Review Board.

### Card study

Card studies are observational studies that collect patient level data at the point of care. They have been used for more than 30 years and have been proven effective for describing clinical problems, management issues and outcomes in primary care settings [27]. The card study method can be used to gather information such as observational phenomena, disease incidence and prevalence, practice methods and patterns and clinical practice behaviors. Pocket-sized cards are carried by practitioners and completed at the point of contact with each patient. Cards should be designed to take no longer than 60 s to complete; they are, in essence, a survey that is to be completed while the practitioner is providing care. The cards are designed so the providers can fill them out without having to refer directly to the patient chart [27]. They are used to document, and not to influence, routine care. Although simple in design and implementation, card studies can be valuable in acquiring in-the-moment data about patients, practitioners, and care delivery systems. Card studies are designed to gain information from practitioners who engage primarily in patient care and not in research [27].

### Choice of social factors

The researchers, with members of ATSU SOMA faculty and the ATSU SOMA PBRN, selected a list of 16 SDH factors to include on the card. These factors were developed based on the Institute of Medicine’s (IOM) Committee on the Recommended Social and Behavioral Measures for Electronic Health Records report [13] and the Healthy People 2020 Social Determinants of Health Framework [8] along with consideration of the WHO’s definition of SDH [2] and factors deemed to be important for patients seen in each CHC setting.

After the initial run of the study in California, minor protocol changes were made to better capture SDH factors and improve data collection. For the New York and Illinois sites, three SDH factors were added: Insufficient or Lack of Insurance, Homelessness, and Unstable Housing. These factors were added based on the needs of the communities served by the NY and IL CHCs, where homelessness and insufficient insurance are more common than in the community served by the CA CHC. The CA site is rural and serves a significant number of migrant farm workers. The NY and IL sites are urban. The categories and listing of all SDH factors used in the study are included in Table 1.

**Table 1:** Categorizations of social factors included

Category	Factors
Access to resources	Educational limitations
	Language barrier
	Poverty
	Near poverty
	Insufficient or lack of insurance^a^
	Food insecurity
Lack of autonomy	Homelessness^a^
	Unstable housing^a^
	Unstable work schedule
	Transportation issues
	Family care demands/Issues
Social and home life	Cultural beliefs
	Immigrant status
	Migrant status
	Neighborhood safety
Optional	Other

Table 1 shows the SDH factors included in this study

^a^Factors not included in the California study

The selected factors were chosen to reflect SDH which are not included in current routine social history screening practices. Therefore, behavioral factors, such as alcohol and tobacco use, and psychological domains such as mood and stress, were not included. The option for “other” was included to allow providers the ability to record factors not on the provided list.

### Study settings

This study was performed with three distinct CHC organizations located in California, New York and Illinois. Each CHC organization has a number of clinic sites; select locations were recruited to participate based on the population served, the number or providers at the sites and the interest of the medical directors for each site. The three CHC organizations recruited for this study are all Federally Qualified Health Centers with an established relationship with ATSU SOMA and are members of the National Association of Community Health Centers (NACHC). A description of each CHC network and the number of sites which participated in the study is included in Table 2.

**Table 2:** Descriptions of CHC locations and patient demographics

State	Area type	Patients		Patient demographics	At or below 100 % FPI	Total sites	Sites included in study
California	Rural	127,128	18.4 %	Non-Hispanic White	80.0 %	16	3
			76.7 %	Hispanic/Latino			
			1.5 %	Black/African American			
			2.2 %	Asian			
			0.4 %	Am. Indian/Alaska Native			
			0.3 %	Hawaiian/Pacific Islander			
New York	Urban	119,734	17.8 %	Non-Hispanic White	71.5 %	9	3
			47.2 %	Hispanic/Latino			
			20.2 %	Black/African American			
			9.5 %	Asian			
			0.5 %	Am. Indian/Alaska Native			
			0.3 %	Hawaiian/Pacific Islander			
Illinois	Urban	34,076	7.1 %	Non-Hispanic White	77.4 %	10	6
			23 %	Hispanic/Latino			
			68.7 %	Black/African American			
			2.1 %	Asian			
			0.3 %	Am. Indian/Alaska Native			
			0.0 %	Hawaiian/Pacific Islander			

Table 2 provides descriptions of each of the CHC organizations participating in this study

Data collected from: 2014 Health Center Profile, HRSA [28, 31, 33]

The California CHC network is located in central California and has a patient population which is primarily Hispanic, with 42 % of patients speaking a language other than English [28]. The CHC provides services to a number of migrant, seasonal and undocumented farm workers [29]. Approximately 80 % of the CHC’s patients live at or below 100 % of the federal poverty line. The CHC provides enabling services for patients such as transportation assistance, translation services, health education, and nutritional counseling [28].

The New York CHC organization is located throughout the city of Brooklyn and is one of the largest CHC networks in the nation offering a full range of health and dental care, behavioral health, specialty services and community based programs [30]. Over 71 % of the CHC’s patient population is living at or below 100 % of the federal poverty line and 20.3 % is uninsured. Almost 50 % of the CHC’s patient population is Hispanic and 31.4 % speak a primary language other than English [31].

The Illinois CHC organization is located in Chicago and targets low-income communities throughout the city, which are characterized by an absence of basic services and health care resources [32]. The CHC’s patient population is predominately African American with approximately 77 % of patients living at or below the federal poverty line and 40 % is uninsured [33]. The CHC offers various clinical services, nutrition education, and social support programs [32].

### Recruitment and timeline

Primary care providers from all 3 CHC organizations were recruited through presentations by study Investigators. In total, 43 out of 71 total eligible providers (61 %) consented to participate in the study; 9 out of 12 (75 %) from California, 21 out of 31 (68 %) from New York and 14 out of 28 (50 %) from Illinois.

The active study period was set to run for 4 weeks. In California it ran from May 28th - June 23rd, 2014; in New York from March 5th - April 3rd, 2015; and in Illinois from October 2nd - November 25th, 2015. The study period was extended in Illinois to encourage more participation.

### Survey and education

Upon consent, providers were given a pre-study questionnaire related to the SDH. This 5-item survey was used to assess the following:

1. Providers’ perceptions regarding their familiarity with the SDH concept and how well they feel they can identify SDH for their patients.
2. How important the providers feel SDH are for their patients, and how much the providers feel these factors affect their patients’ health.
3. Provider perceptions of CHC resources to address SDH and how often they refer patients for these services.

The survey items were measured using five options for each response. The questionnaire is exhibited in Additional file 1 - Provider Questionnaire.

Following the survey, the providers were given a brief (20 min) lecture to define the SDH concept and describe the role SDH play in patient health. Additionally, providers were introduced to the study and given training related to the specific “card” used. This included a description of how to fill out the cards and definitions of the SDH factors.

### Card study

After pre-survey activities were completed, providers were asked to participate in the card study in which qualitative data were collected pertaining to their ability to recognize, document and charge reimbursement fees related to the identified SDH. The cards presented a grid with lines for each potential social factor and also provided space for providers to add their opinions regarding how the identified social factors affected each specific patient’s health. The providers were instructed to complete a card for every patient they saw on one designated day each week for the 4-week time period. Providers were instructed to practice and code in their normal manner. They were specifically instructed not to change their routine or to look up specific codes for this study. The cards in this study were designed to assess the following:

1. Common SDH within the CHC’s catchment area.
2. Provider ability to provide counseling or other interventions for identified SDH.
3. Provider impressions related to the health effects of identified SDH.
4. Provider ability to bill for SDH directed interventions.
5. Provider ability to enter SDH specific diagnosis codes.

The card instructions and grids were printed on 9 × 6 in. index cards and were color-coded based on provider specialty (California) or clinic location (New York and Illinois). Cards were to be completed in an anonymous fashion with no identifying information being added about the patients or practitioners. At the end of each assigned day, practitioners placed cards in a designated drop box and the cards were collected by investigators at the end of each week. A copy of the study card is exhibited in Additional file 2 - Study Card.

## Results

### Survey

All 43 of the participating providers completed the survey prior to beginning the card study. Table 3 presents the results of the survey, which indicate the providers were familiar with the SDH concept and were comfortable with identifying SDH at the point of care. The most frequently chosen response for the first two survey items “How familiar are you with the Social Determinants of Health Concept?” and “How comfortable are you identifying Social Determinants at the point of care?” was “Very Familiar.” Additionally, providers agreed that SDH factors contribute to the health of their patients and they often refer patients to CHC resources to address identified SDH. However, most providers were neutral on whether their CHC had adequate resources to address SDH. Overall, providers at the California CHC responded most favorably when describing their understanding and ability to address SDH factors, whereas providers from the Illinois CHC network responded the least favorably.

**Table 3:** Summary of most frequent responses from provider survey

	California	New York	Illinois	Combined
Question				
1. How familiar are you with the SDH concept?	Very familiar	Very familiar	Somewhat familiar	Very familiar
2. How comfortable are you identifying SDH at the point of care?	Very familiar	Very familiar	Somewhat familiar	Very familiar
3. To what extent do you feel social factors contribute to your patient’s medical condition?	Extremely	Very much	Very much	Very much
4. As part of your treatment plan, how often do you refer patients to CHC resources to address SDH?	Often	Sometimes	Often	Often
5. My CHC has adequate resources available to address specific SDH affecting patient health.	Neutral	Neutral	Neutral	Neutral

Table 3 indicates that most providers agreed with being familiar with the SDH concept, that they were comfortable identifying SDH at the point of care and that SDH have an impact on patients’ health. Survey questions each offered 5 options as answers. For items 1 and 2 these were; not familiar, somewhat familiar, neutral, very familiar and extremely familiar. For item 3 they were; not at all, somewhat, neutral, very much and extremely. For item 4 they were; never, rarely, sometimes, often and all of the time. For item 5 they were; strongly disagree, disagree, neutral, agree and strongly agree

### Card study

Among all three CHC networks, there were a total of 747 patient encounters for which cards were completed; 267 in California, 394 in New York and 86 in Illinois. Only 34 patient encounters had no SDH factors identified by providers, with the remaining patient encounters having from 1 to 12 social factors identified. Overall, 1584 SDH factors were identified resulting in an average of 2.12 factors per encounter. Of these identified factors, 493 received counseling and intervention strategies while only 108 diagnosis codes and 20 billing codes were added to patient charts. The most frequently identified factors were Educational Limitations, Language Barriers, and Family Care Demands.

Table 4 provides a summary of SDH factors identified, organized greatest to least. The table also shows the number of services provided per SDH and the percentage of each SDH reported as billed or coded by the providers. Almost one third of the SDH were addressed through provider services (31.1 %) with very few documented with a diagnosis code (6.8 %) and only rarely could providers bill for their services addressing the SDH (1.2 %).

**Table 4:** Summary of social factors identified, billed and coded

Indicator	Count	Provided service	Billing code	Diagnosis code
Educational limitations	234	104 (44.4 %)	2 (0.9 %)	5 (2.1 %)
Language barrier	176	60 (34.1 %)	3 (1.7 %)	3 (1.7 %)
Family care demands	144	67 (46.5 %)	5 (3.5 %)	34 (23.6 %)
Poverty	141	24 (17.0 %)	1 (0.7 %)	5 (3.5 %)
Unstable work schedule	132	21 (15.9 %)	1 (0.8 %)	2 (1.5 %)
Transportation issues	122	51 (41.8 %)	0 (0.0 %)	0 (0.0 %)
Near poverty	99	17 (17.2 %)	1 (1.0 %)	3 (3.0 %)
Lack of insurance^a^	86	15 (17.4 %)	0 (0.0 %)	3 (3.5 %)
Homelessness^a^	68	3 (4.4 %)	0 (0.0 %)	13 (19.1 %)
Food insecurity	67	40 (59.7 %)	1 (1.5 %)	29 (43.3 %)
Other	67	26 (38.8 %)	2 (3.0 %)	2 (3.0 %)
Immigrant status	66	9 (13.6 %)	0 (0.0 %)	1 (1.5 %)
Cultural beliefs	60	24 (40.0 %)	3 (5.0 %)	2 (3.3 %)
Unstable housing^a^	55	6 (10.9 %)	0 (0.0 %)	4 (7.3 %)
Neighborhood safety	48	24 (50.0 %)	0 (0.0 %)	2 (4.2 %)
Migrant status	19	2 (10.5 %)	0 (0.0 %)	0 (0.0 %)
Total	1584	493 (31.1 %)	19 (1.2 %)	108 (6.8 %)

Table 4 shows a summary of the SDH factors identified, billed and coded by providers

^a^These factors were not included in the California study card

The results from the “other” category are detailed in Table 5. The factors listed in the “other” category include substance abuse and other issues frequently included in the social history. These also included behavioral health issues and poor living conditions.

**Table 5:** Factors identified as “Other” by CHC location

CHC location	Categorized factors	Count	Uncategorized factors	Total
California	Drug use (6)	25	9	34
	Tobacco use (6)			
	Poor living conditions (4)			
	Behavioral health issues (3)			
	Difficulty making appointments (2)			
	Health literacy (1)			
	Interrupted care - Separated parents (1)			
	History of incarceration (1)			
	Age (1)			
New York	Behavioral health issues (12)	30	3	33
	Poor social support (8)			
	Work conditions (5)			
	Physical disability (2)			
	Environmental conditions (1)			
	Drug use (1)			
	Drug use in family (1)			
Illinois		0	0	0

Table 5 shows the number of factors identified by providers as “Other” by each CHC location. For each “other” identified, providers were asked to include descriptions which were then categorized into the above factors and tallied. In some cases, “other” factors were marked as identified, but did not receive a description by the provider and therefore could not be categorized

Table 6 shows the distribution of SDH factors identified by CHC organization. There was an average of 2.12 SDH factors identified during each patient encounter. The most reported SDH factor overall was Educational Limitations. This factor was also the most reported at the California and New York sites. The most reported SDH factor in Illinois was Language Barrier.

**Table 6:** Determinants by CHC location

CHC location	SDH count	Encounters	Average/Encounter	Most reported
California	629	267	2.36	Educational limitations
New York	813	394	2.06	Educational limitations
Illinois	142	86	1.65	Language barrier
Total	1584	747	2.12	Educational limitations

Table 6 shows the number of SDH factors reported by each CHC location and the average number of factors observed per patient encounter

As detailed in Table 7, the California CHC network reported the highest rate of the use of codes to bill for the provision of services to address the SDH (10) and for specific SDH diagnoses (68). The SDH factor “Family Care Demands” was found to be the factor for which services rendered were coded most frequently with a billing code (5) and which received the most diagnosis codes (34). Overall, billing code documentation and diagnosis code documentation specific to the SDH were low across all CHC locations.

**Table 7:** SDH factors coded and billed by CHC location

CHC location	Factors coded	Factors billed	Most frequent services billed	Most frequent diagnosis code
California	68 (10.8 %)	10 (1.6 %)	Family care demands (3)	Family care demands (29)
New York	32 (3.9 %)	9 (1.1 %)	Language barrier (2)	Homelessness (13)
			Family care demands (2)	
			Cultural barrier (2)	
Illinois	8 (5.6 %)	1 (0.7 %)	Neighborhood safety (1)	Neighborhood safety (2) Family care demands (2)
Total	108 (6.8 %)	20 (1.3 %)	Family care demands (5)	Family care demands (34)

Table 7 shows the SDH factors coded and billed by CHC site and also presents the SDH factors most frequently billed and coded at each site

## Discussion

Based on the data gathered, all three CHC networks have patient populations that likely would benefit from systems to identify, address and formally account for SDH factors. Although all three CHC networks have enabling services in place to address the most frequently identified SDH, less than half (31 %) of the identified factors were reported to be addressed by providers and only rarely were providers able to code for their services provided to address the social factors with counseling, education or another intervention. Further, very few providers were able to enter diagnosis codes into the EHR for the specifically identified SDH. Although not every provider from the CHC organizations participated in the study, participants reported being familiar with the SDH concept, and they expressed the opinion that it is an important aspect to consider in treating patients.

There is an obvious discrepancy between the number of identifiable SDH factors, provider ability to address the factors, documentation of the providers’ actions to address the factors using billing codes and documentation of the factors using diagnosis codes. While providers are identifying those SDH factors that they perceive to be impacting their patients’ health, they are not able to provide counseling or other interventions for all of the identified factors. This disparity could be related to their inability to code for SDH factors or bill for related time and services. With very few SDH factors documented with diagnosis codes and even fewer tied to reimbursement codes, it is no surprise providers could not address each in a clinic visit.

The International Classification of Diseases, Version 10 (ICD-10) went into effect in the United States on October 1, 2015 and contains 71,924 procedure codes and 69,823 diagnosis codes [34]. Chapter XXI of ICD-10 contains a listing of Z-Codes categorized as “Factors influencing health status and contact with health services”. Included in this chapter of codes is a sub-category of codes (Z55- Z65) intended for “Persons with potential health hazards related to socioeconomic and psychosocial circumstances”. The 11 codes included in this group cover a range of problems including education and literacy, employment, economic circumstances and social environment. Further branching within these categories produces an additional 96 codes [35].

Table 8 presents each SDH factor used in this study along with an ICD-10 code which best corresponds and a description of why the code does or does not fit. SDH factors which did not have a corresponding ICD-10 code include Language Barriers, Insufficient/Lack of Insurance, Transportation Issues, Immigrant Status and Migrant status. Although most factors had a relevant corresponding code, these codes are not SDH-specific and do not fully cover the intended issues. The ICD-9 used the same Z-codes but in a different format, these were still in place for the CA and NY runs of this study. Looking towards the future, the ICD-10 codes are detailed here.

**Table 8:** SDH factors and related ICD-10 codes

SDH factor and definition	Related ICD-10 code	Review of code
Educational limitations	Z55.0 - Illiteracy and low-level literacy	Other corresponding ICD-10 codes were related to children in school. Patient may be lacking in health literacy, but have appropriate level of reading literacy. The available code is too specific.
Observed difficulty processing and understanding medical information. Can include difficulty reading, listening, asking questions or applying information.		
Language barriers	None identified	
Primary language not English; inability to communicate freely and openly with provider.		
Poverty	Z59.5 - Extreme poverty	Relatively good match with the social factor.
Income below poverty line; lack of basic needs such as nutrition, clothing, shelter.		
Near poverty	Z59.6 - Low income	Relatively good match with the social factor.
Just enough money to meet basic needs but not enough for extras. Qualifies for sliding fee discounts at FQHC.		
Insufficient/Lack of insurance	None identified	
Either no health insurance or has insurance which is not sufficient to cover medical expenses or doesn’t cover medications. Prohibits seeking care or follow through.		
Food insecurity	Z59.4 - Lack of adequate food and safe drinking water	ICD-10 code is a good match. It may be advantageous to have additional codes which address food availability separately from ability to afford food.
Does not have reliable access to sufficient quantity of affordable, nutritious food. Does not know where next meal is coming from. Might live in food desert.		
Homeless	Z59.0 - Homelessness	Relatively good match with the social factor.
Does not have permanent housing, may live on the streets, in a shelter, mission, abandoned building, vehicle or any unstable non-permanent situation.		
Unstable housing	Z59.1 - Inadequate housing	Relatively good match with the social factor.
Lives in several places. Lacks fixed, regular and adequate residence due to loss of housing or economic need. No lease, ownership or occupancy agreement. Unsure if place of shelter will be open for extended period of time.		
Unstable work schedule	Z56.3 - Stressful work schedule	ICD-10 code is related, but does not cover a work schedule which may prevent patients from seeking regular medical care.
Difficulty scheduling or keeping appointments due to variable work schedule; multiple jobs, varying start/stop times, long shifts or unsure when will work.		
Transportation issues	None identified	
Hard to get to appointments due to lack of transportation. Does not own vehicle, can’t afford public transportation, lives far from public transportation or services unreliable.		
Family care demands/Issues	Z63.2 - Inadequate family support	ICD-10 code is related, but somewhat different; does not cover patients who are unable to care for themselves due to the demands of family care.
	Z63.6 - Dependent relative needing care at home	
Responsibilities at home caring for others (children, partner, parents, family) which prevent person from caring for themselves.		
Cultural beliefs	Z60.3 - Acculturation difficulty	ICD-10 code is somewhat related but doesn’t specifically cover issues pertaining to cultural beliefs which may prohibit a patient from seeking or following medical advice.
Cultural background is not in concordance with Western Medicine. May believe Western Medicine can be detrimental or is the place of last resort. Beliefs may conflict with medical care - prohibit patient from seeking care or adhering to treatment plan.		
Immigrant status	None identified	
Not born in US, now living here legally or illegally. Can have difficulty obtaining public assistance if ‘illegal’. May be child with legal status whose parents do not have legal status.		
Migrant status	None identified	
Person is a migrant worker who relocates frequently due to work availability.		
Neighborhood safety	Z59.2 - Discord with Neighbors, lodgers, landlord	ICD-10 code is related but there remains a lack of codes which address safety and environmental issues. The available code does not clearly match the issue.
Not feel safe going outside in neighborhood, threat of crime/violence. Under stress from environment. Children can’t play outside, can’t exercise, hard to get to appointments.		

Table 8 provides a listing of the SDH factors used by investigators for this study along with related ICD-10 codes. The last column provides a description of how well the codes are matched

It is unclear whether the CHC providers are familiar with these coding options and whether they find them useful for SDH factors. However, due to the low rate of recorded codes, it appears these codes are under-utilized and may not be easily documented in an EHR. Tools or procedures to improve the documentation and monitoring of SDH factors need to be considered, such as SDH-specific screening tools or the addition of SDH factors to standard vital signs. Further, codes do not exist for many of the identified SDH and those that do exist are frequently pointing to different issues than the specific SDH providers observed in this study.

### Limitations

This study was performed at three separate CHC organizations in California, New York and Illinois with a total of 43 providers who together documented 747 patient visits. This study was limited through its small size and lack of generalizability. Providers volunteered to participate and could represent CHC providers who are more inclined towards recognizing the SDH. Only a portion of overall patient visits over a defined time period were documented on the cards. Thus, this study represents only a snapshot of what is happening at the point of care.

Given the study design with anonymous cards (for both the providers and patients) we can not determine the proportion of patients seen by any given provider for which a card was filled out. The average number of cards per provider was 17. This is less than the number of patients an average provider would see on 4 clinic days. Thus we cannot generalize the average number of SDH per patient to all CHC patients. Nevertheless, obvious discrepancies in SDH identified and accounted for are evident.

## Conclusions

The current state of the health care system coupled with wide spread expansion of coverage provides CHCs with an excellent opportunity to influence change in the way health care is delivered and the focus that is placed on actions at the point of care. This card study is easily replicable, and could be performed in any ambulatory care practice setting to determine providers’ perceptions regarding what SDH issues the patient populations are facing and the ways in which providers are documenting and addressing these issues. PBRN leaders are encouraged to contact the principal investigator in order to replicate this study.

It is suggested that the current infrastructure is deficient in that it lacks the “systems put in place to deal with illness”, as recommended by the WHO definition of SDH [2]. While data indicate awareness on the part of CHC providers regarding the SDH, their inability to bill for services rendered to address the SDH and inability to enter diagnosis codes for the specific SDH undermines the goals of the ACA. While there are diagnosis codes available, which are somewhat related to 10 of the SDH identified in this study, only 4 of the ICD-10 codes were deemed a good match with the SDH by the study team. Further work should explore why the available codes were not used. It is striking that these codes were not used by a population of providers who express familiarity with the SDH and who specifically work at CHCs where service is provided regardless of ability to pay. Future research should also explore provider interpretations of the present codes and suggestions for additional codes. Education programs to increase the use of SDH- related codes could be implemented and evaluated.

Improving the US health care system and the public’s health must begin with attention to all factors impacting an individual’s or a group of individuals’ health. The IOM’s new framework for educating health professionals to address the social determinants of health is a good start [5]. The ACA expanded coverage to more Americans, can also help. However, coverage in and of itself does not address social barriers to care or social factors affecting health and well-being. Providers must be given the tools within the emerging systems to code, bill and be reimbursed as they seek to address the SDH negatively affecting their patients.

CHCs and other health care organizations should seek to implement procedures to document and monitor SDH and the actions taken to address them. The IOM developed EHR screening tool is one available resource intended to allow providers an efficient way to screen and follow-up on identified SDH issues [10, 13]. While possibly related, The SDH factors included in the IOM tool vary from those used in this study. While the IOM screening tool includes behavioral and psychosocial factors not included in this study, the IOM factors are not comprehensive or specific to the detailed SDH factors identified by study providers.

The results of this study suggest simple methods of SDH identification may be sufficient, as providers are able to check off identified factors after patient encounters. With minimal direction and the use of a simple tool such as the study card, providers were able to identify SDH through normal clinical practice and conversations with their patients. SDH factors could be included in EHRs along with standard vital signs and marked when identified during a patient encounter. The identified SDH could then be automatically linked with diagnosis codes. Further EHR solutions could automate the linkage to billing codes for actions taken to address the specific SDH. Allowing the potential to receive reimbursement for SDH specific procedures could potentially increase the rates at which providers address these issues.

The implementation of any new tool or procedure comes with the potential for an increased burden on providers and health organizations. Therefore, attention needs to be paid to ensuring an efficient system. The SDH factors utilized in this study could also be assessed through patient self-report obtained during a normal clinical encounter. The use of a simple check-list could potentially serve as an effective mode of documentation. The results of the study indicate providers are already identifying SDH factors affecting their patients and spending time during clinical encounters to address these factors. With the addition of searchable codes and reimbursements, we envision improvements in the way these factors are addressed on both an individual and a population level.

A growing number of providers, public health and policy experts are working towards expanding standard vital signs to include specific SDH [10, 13, 17]. Only when the SDH are incorporated into the EHR can they be quantified, reviewed and fully addressed by health care providers and health systems. Including the SDH in the EHR can enable more effective treatment of patients at the point of care and more effective population management. Knowledge is power, and in the EHR world, the knowledge must be available in a systematic and searchable mechanism. With the ability to directly tie observed SDH factors to health outcomes for individuals and populations, future funds and programs can more efficiently and more effectively promote true health.

## Additional files

Additional file 1: Provider questionnaire. Description of file: File contains the questionnaire completed by participating providers. (DOCX 15 kb) Additional file 2: Study card. Description of file: File provides an example of the card used for the study. (DOCX 28 kb) Additional file 3: Card data by location. Description of data: Data is broken down by location to show each card that was completed. Each CHC site has two corresponding data sheets. The first sheet contains the raw data including totals which show how many SDH was recorded. The second sheet contains tables with counts for each SDH and how often it was billed and/or diagnosed. (XLSX 235 kb) Additional file 4: Survey data by location. Description of data: Coded survey responses for each provider are included by CHC location. For each question, the average score and mode are provided. A key is included which shows the answer for each coded score. (XLSX 14 kb)

## Abbreviations

ACA: Patient Protection and Affordable Care Act
ATSU SOMA: A.T. Still University of Health Sciences School of Osteopathic Medicine in Arizona
ATSU SOMA PBRN: A.T. Still University School of Osteopathic Medicine in Arizona Practice-Based Research Network
CHC: Community Health Center
EHR: Electronic Health Record
ICD-10: International Classification of Diseases, Volume 10
IOM: Institute of Medicine
NACHC: National Association of Community Health Centers
SDH: Social determinants of health
WHO: World Health Organization
