# Supplementary material for: Community health center provider ability to identify, treat and account for the social determinants of health: a card study
Source: BMC Fam Pract. 2016 Aug 27;17(1):121. doi: 10.1186/s12875-016-0526-8 (PMC5002327; doi:10.1186/s12875-016-0526-8)
Supplement: Additional file 1: — Provider questionnaire. Description of file: File contains the questionnaire completed by participating providers. (DOCX 15 kb) [file 12875_2016_526_MOESM1_ESM.docx]

Provider Questionnaire

**1. How familiar are you with the Social Determinants of Health Concept?**

| Not  Familiar | Somewhat Familiar | Neutral | Very  Familiar | Extremely Familiar |
| --- | --- | --- | --- | --- |

**2. How comfortable are you identifying Social Determinants at the point of care?**

| Not Comfortable | Somewhat Comfortable | Neutral | Very Comfortable | Extremely Comfortable |
| --- | --- | --- | --- | --- |

**3. To what extent do you feel social factors contribute to your patients’ medical conditions?**

| Not At All | Somewhat | Neutral | Very Much | Extremely |
| --- | --- | --- | --- | --- |

**4. As part of your treatment plan, how often do you refer patients to CHC resources to**

**address social determinants of health?**

| Never | Rarely | Sometimes | Often | All of the Time |
| --- | --- | --- | --- | --- |

**5. My CHC has adequate resources available to address specific social determinants affecting**

**my patients’ health.**

| Strongly Disagree | Disagree | Neutral | Agree | Strongly Agree |
| --- | --- | --- | --- | --- |
