# Supplementary material for: Community health center provider ability to identify, treat and account for the social determinants of health: a card study
Source: BMC Fam Pract. 2016 Aug 27;17(1):121. doi: 10.1186/s12875-016-0526-8 (PMC5002327; doi:10.1186/s12875-016-0526-8)
Supplement: Additional file 2: — Study card. Description of file: File provides an example of the card used for the study. (DOCX 28 kb) [file 12875_2016_526_MOESM2_ESM.docx]

Study Card

Front:

Back:
